# Search for Higgs-like bosons decaying into long-lived exotic particles

**DOI:** 10.1140/epjc/s10052-016-4489-7

**Published:** 2016-12-02

**Authors:** R. Aaij, B. Adeva, M. Adinolfi, Z. Ajaltouni, S. Akar, J. Albrecht, F. Alessio, M. Alexander, S. Ali, G. Alkhazov, P. Alvarez Cartelle, A. A. Alves, S. Amato, S. Amerio, Y. Amhis, L. An, L. Anderlini, G. Andreassi, M. Andreotti, J. E. Andrews, R. B. Appleby, O. Aquines Gutierrez, F. Archilli, P. d’Argent, J. Arnau Romeu, A. Artamonov, M. Artuso, E. Aslanides, G. Auriemma, M. Baalouch, S. Bachmann, J. J. Back, A. Badalov, C. Baesso, W. Baldini, R. J. Barlow, C. Barschel, S. Barsuk, W. Barter, V. Batozskaya, V. Battista, A. Bay, L. Beaucourt, J. Beddow, F. Bedeschi, I. Bediaga, L. J. Bel, V. Bellee, N. Belloli, K. Belous, I. Belyaev, E. Ben-Haim, G. Bencivenni, S. Benson, J. Benton, A. Berezhnoy, R. Bernet, A. Bertolin, M.-O. Bettler, M. van Beuzekom, I. Bezshyiko, S. Bifani, P. Billoir, T. Bird, A. Birnkraut, A. Bitadze, A. Bizzeti, T. Blake, F. Blanc, J. Blouw, S. Blusk, V. Bocci, T. Boettcher, A. Bondar, N. Bondar, W. Bonivento, S. Borghi, M. Borisyak, M. Borsato, F. Bossu, M. Boubdir, T. J. V. Bowcock, E. Bowen, C. Bozzi, S. Braun, M. Britsch, T. Britton, J. Brodzicka, E. Buchanan, C. Burr, A. Bursche, J. Buytaert, S. Cadeddu, R. Calabrese, M. Calvi, M. Calvo Gomez, P. Campana, D. Campora Perez, L. Capriotti, A. Carbone, G. Carboni, R. Cardinale, A. Cardini, P. Carniti, L. Carson, K. Carvalho Akiba, G. Casse, L. Cassina, L. Castillo Garcia, M. Cattaneo, Ch. Cauet, G. Cavallero, R. Cenci, M. Charles, Ph. Charpentier, G. Chatzikonstantinidis, M. Chefdeville, S. Chen, S.-F. Cheung, V. Chobanova, M. Chrzaszcz, X. Cid Vidal, G. Ciezarek, P. E. L. Clarke, M. Clemencic, H. V. Cliff, J. Closier, V. Coco, J. Cogan, E. Cogneras, V. Cogoni, L. Cojocariu, G. Collazuol, P. Collins, A. Comerma-Montells, A. Contu, A. Cook, S. Coquereau, G. Corti, M. Corvo, C. M. Costa Sobral, B. Couturier, G. A. Cowan, D. C. Craik, A. Crocombe, M. Cruz Torres, S. Cunliffe, R. Currie, C. D’Ambrosio, E. Dall’Occo, J. Dalseno, P. N. Y. David, A. Davis, O. De Aguiar Francisco, K. De Bruyn, S. De Capua, M. De Cian, J. M. De Miranda, L. De Paula, P. De Simone, C.-T. Dean, D. Decamp, M. Deckenhoff, L. Del Buono, M. Demmer, D. Derkach, O. Deschamps, F. Dettori, B. Dey, A. Di Canto, H. Dijkstra, F. Dordei, M. Dorigo, A. Dosil Suárez, A. Dovbnya, K. Dreimanis, L. Dufour, G. Dujany, K. Dungs, P. Durante, R. Dzhelyadin, A. Dziurda, A. Dzyuba, N. Déléage, S. Easo, U. Egede, V. Egorychev, S. Eidelman, S. Eisenhardt, U. Eitschberger, R. Ekelhof, L. Eklund, Ch. Elsasser, S. Ely, S. Esen, H. M. Evans, T. Evans, A. Falabella, N. Farley, S. Farry, R. Fay, D. Ferguson, V. Fernandez Albor, F. Ferrari, F. Ferreira Rodrigues, M. Ferro-Luzzi, S. Filippov, M. Fiore, M. Fiorini, M. Firlej, C. Fitzpatrick, T. Fiutowski, F. Fleuret, K. Fohl, M. Fontana, F. Fontanelli, D. C. Forshaw, R. Forty, M. Frank, C. Frei, M. Frosini, J. Fu, E. Furfaro, C. Färber, A. Gallas Torreira, D. Galli, S. Gallorini, S. Gambetta, M. Gandelman, P. Gandini, Y. Gao, J. García Pardiñas, J. Garra Tico, L. Garrido, P. J. Garsed, D. Gascon, C. Gaspar, L. Gavardi, G. Gazzoni, D. Gerick, E. Gersabeck, M. Gersabeck, T. Gershon, Ph. Ghez, S. Gianì, V. Gibson, O. G. Girard, L. Giubega, K. Gizdov, V. V. Gligorov, D. Golubkov, A. Golutvin, A. Gomes, I. V. Gorelov, C. Gotti, M. Grabalosa Gándara, R. Graciani Diaz, L. A. Granado Cardoso, E. Graugés, E. Graverini, G. Graziani, A. Grecu, P. Griffith, L. Grillo, B. R. Gruberg Cazon, O. Grünberg, E. Gushchin, Yu. Guz, T. Gys, C. Göbel, T. Hadavizadeh, C. Hadjivasiliou, G. Haefeli, C. Haen, S. C. Haines, S. Hall, B. Hamilton, X. Han, S. Hansmann-Menzemer, N. Harnew, S. T. Harnew, J. Harrison, J. He, T. Head, A. Heister, K. Hennessy, P. Henrard, L. Henry, J. A. Hernando Morata, E. van Herwijnen, M. Heß, A. Hicheur, D. Hill, C. Hombach, W. Hulsbergen, T. Humair, M. Hushchyn, N. Hussain, D. Hutchcroft, M. Idzik, P. Ilten, R. Jacobsson, A. Jaeger, J. Jalocha, E. Jans, A. Jawahery, M. John, D. Johnson, C. R. Jones, C. Joram, B. Jost, N. Jurik, S. Kandybei, W. Kanso, M. Karacson, J. M. Kariuki, S. Karodia, M. Kecke, M. Kelsey, I. R. Kenyon, M. Kenzie, T. Ketel, E. Khairullin, B. Khanji, C. Khurewathanakul, T. Kirn, S. Klaver, K. Klimaszewski, S. Koliiev, M. Kolpin, I. Komarov, R. F. Koopman, P. Koppenburg, A. Kozachuk, M. Kozeiha, L. Kravchuk, K. Kreplin, M. Kreps, P. Krokovny, F. Kruse, W. Krzemien, W. Kucewicz, M. Kucharczyk, V. Kudryavtsev, A. K. Kuonen, K. Kurek, T. Kvaratskheliya, D. Lacarrere, G. Lafferty, A. Lai, D. Lambert, G. Lanfranchi, C. Langenbruch, B. Langhans, T. Latham, C. Lazzeroni, R. Le Gac, J. van Leerdam, J.-P. Lees, A. Leflat, J. Lefrançois, R. Lefèvre, F. Lemaitre, E. Lemos Cid, O. Leroy, T. Lesiak, B. Leverington, Y. Li, T. Likhomanenko, R. Lindner, C. Linn, F. Lionetto, B. Liu, X. Liu, D. Loh, I. Longstaff, J. H. Lopes, D. Lucchesi, M. Lucio Martinez, H. Luo, A. Lupato, E. Luppi, O. Lupton, A. Lusiani, X. Lyu, F. Machefert, F. Maciuc, O. Maev, K. Maguire, S. Malde, A. Malinin, T. Maltsev, G. Manca, G. Mancinelli, P. Manning, J. Maratas, J. F. Marchand, U. Marconi, C. Marin Benito, P. Marino, J. Marks, G. Martellotti, M. Martin, M. Martinelli, D. Martinez Santos, F. Martinez Vidal, D. Martins Tostes, L. M. Massacrier, A. Massafferri, R. Matev, A. Mathad, Z. Mathe, C. Matteuzzi, A. Mauri, B. Maurin, A. Mazurov, M. McCann, J. McCarthy, A. McNab, R. McNulty, B. Meadows, F. Meier, M. Meissner, D. Melnychuk, M. Merk, E. Michielin, D. A. Milanes, M.-N. Minard, D. S. Mitzel, J. Molina Rodriguez, I. A. Monroy, S. Monteil, M. Morandin, P. Morawski, A. Mordà, M. J. Morello, J. Moron, A. B. Morris, R. Mountain, F. Muheim, M. Mulder, M. Mussini, D. Müller, J. Müller, K. Müller, V. Müller, P. Naik, T. Nakada, R. Nandakumar, A. Nandi, I. Nasteva, M. Needham, N. Neri, S. Neubert, N. Neufeld, M. Neuner, A. D. Nguyen, C. Nguyen-Mau, V. Niess, S. Nieswand, R. Niet, N. Nikitin, T. Nikodem, A. Novoselov, D. P. O’Hanlon, A. Oblakowska-Mucha, V. Obraztsov, S. Ogilvy, R. Oldeman, C. J. G. Onderwater, J. M. Otalora Goicochea, A. Otto, P. Owen, A. Oyanguren, A. Palano, F. Palombo, M. Palutan, J. Panman, A. Papanestis, M. Pappagallo, L. L. Pappalardo, C. Pappenheimer, W. Parker, C. Parkes, G. Passaleva, G. D. Patel, M. Patel, C. Patrignani, A. Pearce, A. Pellegrino, G. Penso, M. Pepe Altarelli, S. Perazzini, P. Perret, L. Pescatore, K. Petridis, A. Petrolini, A. Petrov, M. Petruzzo, E. Picatoste Olloqui, B. Pietrzyk, M. Pikies, D. Pinci, A. Pistone, A. Piucci, S. Playfer, M. Plo Casasus, T. Poikela, F. Polci, A. Poluektov, I. Polyakov, E. Polycarpo, G. J. Pomery, A. Popov, D. Popov, B. Popovici, C. Potterat, E. Price, J. D. Price, J. Prisciandaro, A. Pritchard, C. Prouve, V. Pugatch, A. Puig Navarro, G. Punzi, W. Qian, R. Quagliani, B. Rachwal, J. H. Rademacker, M. Rama, M. Ramos Pernas, M. S. Rangel, I. Raniuk, G. Raven, F. Redi, S. Reichert, A. C. dos Reis, C. Remon Alepuz, V. Renaudin, S. Ricciardi, S. Richards, M. Rihl, K. Rinnert, V. Rives Molina, P. Robbe, A. B. Rodrigues, E. Rodrigues, J. A. Rodriguez Lopez, P. Rodriguez Perez, A. Rogozhnikov, S. Roiser, V. Romanovskiy, A. Romero Vidal, J. W. Ronayne, M. Rotondo, T. Ruf, P. Ruiz Valls, J. J. Saborido Silva, N. Sagidova, B. Saitta, V. Salustino Guimaraes, C. Sanchez Mayordomo, B. Sanmartin Sedes, R. Santacesaria, C. Santamarina Rios, M. Santimaria, E. Santovetti, A. Sarti, C. Satriano, A. Satta, D. M. Saunders, D. Savrina, S. Schael, M. Schiller, H. Schindler, M. Schlupp, M. Schmelling, T. Schmelzer, B. Schmidt, O. Schneider, A. Schopper, M. Schubiger, M.-H. Schune, R. Schwemmer, B. Sciascia, A. Sciubba, A. Semennikov, A. Sergi, N. Serra, J. Serrano, L. Sestini, P. Seyfert, M. Shapkin, I. Shapoval, Y. Shcheglov, T. Shears, L. Shekhtman, V. Shevchenko, A. Shires, B. G. Siddi, R. Silva Coutinho, L. Silva de Oliveira, G. Simi, M. Sirendi, N. Skidmore, T. Skwarnicki, E. Smith, I. T. Smith, J. Smith, M. Smith, H. Snoek, M. D. Sokoloff, F. J. P. Soler, D. Souza, B. Souza De Paula, B. Spaan, P. Spradlin, S. Sridharan, F. Stagni, M. Stahl, S. Stahl, P. Stefko, S. Stefkova, O. Steinkamp, O. Stenyakin, S. Stevenson, S. Stoica, S. Stone, B. Storaci, S. Stracka, M. Straticiuc, U. Straumann, L. Sun, W. Sutcliffe, K. Swientek, V. Syropoulos, M. Szczekowski, T. Szumlak, S. T’Jampens, A. Tayduganov, T. Tekampe, G. Tellarini, F. Teubert, C. Thomas, E. Thomas, J. van Tilburg, V. Tisserand, M. Tobin, S. Tolk, L. Tomassetti, D. Tonelli, S. Topp-Joergensen, E. Tournefier, S. Tourneur, K. Trabelsi, M. Traill, M. T. Tran, M. Tresch, A. Trisovic, A. Tsaregorodtsev, P. Tsopelas, A. Tully, N. Tuning, A. Ukleja, A. Ustyuzhanin, U. Uwer, C. Vacca, V. Vagnoni, S. Valat, G. Valenti, A. Vallier, R. Vazquez Gomez, P. Vazquez Regueiro, S. Vecchi, M. van Veghel, J. J. Velthuis, M. Veltri, G. Veneziano, A. Venkateswaran, M. Vesterinen, B. Viaud, D. Vieira, M. Vieites Diaz, X. Vilasis-Cardona, V. Volkov, A. Vollhardt, B. Voneki, D. Voong, A. Vorobyev, V. Vorobyev, C. Voß, J. A. de Vries, C. Vázquez Sierra, R. Waldi, C. Wallace, R. Wallace, J. Walsh, J. Wang, D. R. Ward, H. M. Wark, N. K. Watson, D. Websdale, A. Weiden, M. Whitehead, J. Wicht, G. Wilkinson, M. Wilkinson, M. Williams, M. P. Williams, M. Williams, T. Williams, F. F. Wilson, J. Wimberley, J. Wishahi, W. Wislicki, M. Witek, G. Wormser, S. A. Wotton, K. Wraight, S. Wright, K. Wyllie, Y. Xie, Z. Xing, Z. Xu, Z. Yang, H. Yin, J. Yu, X. Yuan, O. Yushchenko, M. Zangoli, K. A. Zarebski, M. Zavertyaev, L. Zhang, Y. Zhang, Y. Zhang, A. Zhelezov, Y. Zheng, A. Zhokhov, V. Zhukov, S. Zucchelli

**Affiliations:** 1 Centro Brasileiro de Pesquisas Físicas (CBPF), Rio de Janeiro, Brazil; 2 Universidade Federal do Rio de Janeiro (UFRJ), Rio de Janeiro, Brazil; 3 Center for High Energy Physics, Tsinghua University, Beijing, China; 4 LAPP, Université Savoie Mont-Blanc, CNRS/IN2P3, Annecy-Le-Vieux, France; 5 Clermont Université, Université Blaise Pascal, CNRS/IN2P3, LPC, Clermont-Ferrand, France; 6 CPPM, Aix-Marseille Université, CNRS/IN2P3, Marseille, France; 7 LAL, Université Paris-Sud, CNRS/IN2P3, Orsay, France; 8 LPNHE, Université Pierreet Marie Curie, Université Paris Diderot, CNRS/IN2P3, Paris, France; 9 I. Physikalisches Institut, RWTH Aachen University, Aachen, Germany; 10 Fakultät Physik, Technische Universität Dortmund, Dortmund, Germany; 11 Max-Planck-Institut für Kernphysik (MPIK), Heidelberg, Germany; 12 Physikalisches Institut, Ruprecht-Karls-Universität Heidelberg, Heidelberg, Germany; 13 School of Physics, University College Dublin, Dublin, Ireland; 14 Sezione INFN di Bari, Bari, Italy; 15 Sezione INFN di Bologna, Bologna, Italy; 16 Sezione INFN di Cagliari, Cagliari, Italy; 17 Sezione INFN di Ferrara, Ferrara, Italy; 18 Sezione INFN di Firenze, Florence, Italy; 19 Laboratori Nazionali dell’INFN di Frascati, Frascati, Italy; 20 Sezione INFN di Genova, Genoa, Italy; 21 Sezione INFN di Milano Bicocca, Milan, Italy; 22 Sezione INFN di Milano, Milan, Italy; 23 Sezione INFN di Padova, Padua, Italy; 24 Sezione INFN di Pisa, Pisa, Italy; 25 Sezione INFN di Roma Tor Vergata, Rome, Italy; 26 Sezione INFN di Roma La Sapienza, Rome, Italy; 27 Henryk Niewodniczanski Institute of Nuclear Physics Polish Academy of Sciences, Kraków, Poland; 28 Faculty of Physics and Applied Computer Science, AGH, University of Science and Technology, Kraków, Poland; 29 National Center for Nuclear Research (NCBJ), Warsaw, Poland; 30 Horia Hulubei National Institute of Physics and Nuclear Engineering, Bucharest-Magurele, Romania; 31 Petersburg Nuclear Physics Institute (PNPI), Gatchina, Russia; 32 Institute of Theoretical and Experimental Physics (ITEP), Moscow, Russia; 33 Institute of Nuclear Physics, Moscow State University (SINP MSU), Moscow, Russia; 34 Institute for Nuclear Research of the Russian Academy of Sciences (INR RAN), Moscow, Russia; 35 Budker Institute of Nuclear Physics (SB RAS), Novosibirsk State University, Novosibirsk, Russia; 36 Institute for High Energy Physics (IHEP), Protvino, Russia; 37 Universitat de Barcelona, Barcelona, Spain; 38 Universidad de Santiago de Compostela, Santiago de Compostela, Spain; 39 European Organization for Nuclear Research (CERN), Geneva, Switzerland; 40 Ecole Polytechnique Fédérale de Lausanne (EPFL), Lausanne, Switzerland; 41 Physik-Institut, Universität Zürich, Zurich, Switzerland; 42 Nikhef National Institute for Subatomic Physics, Amsterdam, The Netherlands; 43 Nikhef National Institute for Subatomic Physics, VU University Amsterdam, Amsterdam, The Netherlands; 44 NSC Kharkiv Institute of Physics and Technology (NSC KIPT), Kharkiv, Ukraine; 45 Institute for Nuclear Research of the National Academy of Sciences (KINR), Kyiv, Ukraine; 46 University of Birmingham, Birmingham, UK; 47 H.H. Wills Physics Laboratory, University of Bristol, Bristol, UK; 48 Cavendish Laboratory, University of Cambridge, Cambridge, UK; 49 Department of Physics, University of Warwick, Coventry, UK; 50 STFC Rutherford Appleton Laboratory, Didcot, UK; 51 School of Physics and Astronomy, University of Edinburgh, Edinburgh, UK; 52 School of Physics and Astronomy, University of Glasgow, Glasgow, UK; 53 Oliver Lodge Laboratory, University of Liverpool, Liverpool, UK; 54 Imperial College London, London, UK; 55 School of Physics and Astronomy, University of Manchester, Manchester, UK; 56 Department of Physics, University of Oxford, Oxford, UK; 57 Massachusetts Institute of Technology, Cambridge, MA USA; 58 University of Cincinnati, Cincinnati, OH USA; 59 University of Maryland, College Park, MD USA; 60 Syracuse University, Syracuse, NY USA; 61 Pontifícia Universidade Católica do Rio de Janeiro (PUC-Rio), Rio de Janeiro, Brazil; 62 University of Chinese Academy of Sciences, Beijing, China; 63 Institute of Particle Physics, Central China Normal University, Wuhan, Hubei China; 64 Departamento de Fisica, Universidad Nacional de Colombia, Bogota, Colombia; 65 Institut für Physik, Universität Rostock, Rostock, Germany; 66 National Research Centre Kurchatov Institute, Moscow, Russia; 67 Yandex School of Data Analysis, Moscow, Russia; 68 Instituto de Fisica Corpuscular (IFIC), Universitat de Valencia-CSIC, Valencia, Spain; 69 Van Swinderen Institute, University of Groningen, Groningen, The Netherlands

## Abstract

A search is presented for massive long-lived particles, in the 20–60 ${\mathrm {Ge V\!/}c^2}$ mass range with lifetimes between 5 and 100 $\mathrm{ps}$. The dataset used corresponds to 0.62$\text{ fb }^{-1}$ of proton-proton collision data collected by the LHCb detector at $\sqrt{s} =7\mathrm {\,Te V}$. The particles are assumed to be pair-produced by the decay of a Higgs-like boson with mass between 80 and 140 ${\mathrm {Ge V\!/}c^2}$. No excess above the background expectation is observed and limits are set on the production cross-section as a function of the long-lived particle mass and lifetime and of the Higgs-like boson mass.

## Introduction

The standard model of particle physics (SM) has shown great success in describing physics processes at very short distances. Nevertheless, open questions remain, such as the hierarchy problem, the imprecise unification of gauge couplings, and the absence of candidates for dark matter. Considerable efforts have been made to address these issues, resulting in a large variety of models. Supersymmetry (SUSY), in which the strong and electroweak forces are unified at a renormalisation scale near the Planck scale, provides a possible solution for the hierarchy problem; the minimal supersymmetric standard model (MSSM) is the simplest, phenomenologically viable realisation of SUSY [1, 2].

The present study focuses on a subset of models featuring massive long-lived particles (LLP) with a measurable flight distance. We concentrate on scenarios in which the LLP decays hadronically in the LHCb vertex detector, travelling distances which can be larger than those of typical $$b $$ hadrons.

A large number of LLP searches have been performed by the experiments at the LHC and Tevatron, mainly using the Hidden Valley framework [3] as a benchmark model [4–8]. Hidden Valley processes have also been sought by LHCb [9], which is able to explore the forward rapidity region only partially covered by other LHC experiments. In addition, it is able to trigger on particles with low transverse momenta, allowing the experiment to probe relatively small LLP masses.

The event topology considered in this study is quite different from that of Hidden Valley models. The minimal supergravity model (mSUGRA) realisation of the MSSM is used as a benchmark model with baryon number violation [10], as suggested in Refs. [11, 12]. Here a Higgs-like boson produced in *pp* collisions decays into two LLPs (neutralinos), subsequently decaying into three quarks each. The Higgs-like particle mass ranges from 80 up to 140 $${\mathrm {Ge V\!/}c^2}$$, covering the mass of the scalar boson discovered by the ATLAS and CMS experiments [13, 14]. The explored LLP lifetime range of 5–100 $$\mathrm{ps}$$ is higher than the typical $$b $$ hadron lifetime, and corresponds to an average flight distance of up to 30 cm, which is inside the LHCb vertex detector region. The LLP mass range considered is between 20 and 60 $${\mathrm {Ge V\!/}c^2}$$.

## Detector description

The LHCb detector [15, 16] is a single-arm forward spectrometer covering the pseudorapidity range $$2<\eta <5$$, designed for the study of particles containing $$b $$ or $$c $$ quarks. The detector includes a high-precision tracking system consisting of a silicon-strip vertex detector surrounding the *pp* interaction region (VELO), a large-area silicon-strip detector located upstream of a dipole magnet with a bending power of about $$4{\mathrm {\,Tm}}$$, and three stations of silicon-strip detectors and straw drift tubes, placed downstream of the magnet. The tracking system provides a measurement of the momentum, $$p$$, of charged particles with a relative uncertainty that varies from 0.5% at low momentum to 1.0% at 200 $${\mathrm {Ge V\!/}c}$$. The minimum distance of a track to a primary vertex (PV), the impact parameter, is measured with a resolution of (15 $$+$$ 29/$$p_\mathrm{T}$$) $$\upmu \mathrm{m}$$, where $$p_\mathrm{T}$$is the component of the momentum transverse to the beam, in $${\mathrm {Ge V\!/}c}$$. Different types of charged hadrons are distinguished using information from two ring-imaging Cherenkov detectors. Photons, electrons and hadrons are identified by a calorimeter system consisting of scintillating-pad and preshower detectors, an electromagnetic calorimeter and a hadronic calorimeter. Muons are identified by a system composed of alternating layers of iron and multiwire proportional chambers. The online event selection is performed by a trigger [17], which consists of a hardware stage, L0, based on information from the calorimeter and muon systems, followed by two software stages, HLT1 and HLT2, which run a simplified version of the offline event reconstruction.

## Event generation and detector simulation

Various simulated event samples are used in this analysis. The *pp* collisions are generated with Pythia  6 [18]. The process simulated is $${\mathrm {h}^0} \rightarrow {\tilde{\chi }^{0}_{1}}{\tilde{\chi }^{0}_{1}}$$, where the Higgs-like boson of mass $$m_\mathrm{h^0}$$ is produced via gluon-gluon fusion, with the parton density function taken from CTEQ6L [19]. The neutralino $$\tilde{\chi }^{0}_{1}$$ is an LLP of mass $$m_\mathrm{{LLP}}$$ and lifetime $$\tau _\mathrm{{LLP}}$$, which decays into three quarks via the mSUGRA baryon number violating process available in Pythia. The corresponding decay flavour structure for the neutralino with a mass of $$48\,{\mathrm {Ge V\!/}c^2} $$ is 18.5% for each of the combinations with a $$b $$ quark (*udb*, *usb*, *cdb*, *csb*), and 13% for each *udq* and *cdq*, where *q* is not a $$b $$ quark, i.e. about 75% of LLPs have a $$b $$ quark in the decay. This fraction becomes 70% for $$m_\mathrm{{LLP}} =20\,{\mathrm {Ge V\!/}c^2} $$.

Two separate detector simulations are used, a full simulation where the interaction of the generated particles with the detector is based on Geant4  [20, 21], and a fast simulation. In Geant4, the detector and its response are implemented as described in Ref. [22]. Signal models for a representative set of theoretical parameters have been generated and fully simulated (Appendix A, Table 5). In the remainder of this paper, the following nomenclature is chosen: a prefix “BV”, indicating baryon number violation, is followed by the LLP mass in ${\mathrm {Ge V\!/}c^2}$ and lifetime, and the prefix “mH” followed by the $m_\mathrm{h^0}$ value in ${\mathrm {Ge V\!/}c^2}$. Most of the fully simulated models have $m_\mathrm{h^0}$=114 ${\mathrm {Ge V\!/}c^2}$, which is in the middle of the chosen Higgs-like particle mass range. Only events with at least one $\tilde{\chi }^{0}_{1}$ in the pseudorapidity region $1.8<\eta <5.0$ are processed by Geant4, corresponding to about 30% of the generated events.

The fast simulation is used to cover a broader parameter space of the theoretical models. Here the charged particles from the ${\mathrm {h}^0} \rightarrow {\tilde{\chi }^{0}_{1}} {\tilde{\chi }^{0}_{1}}$ process falling in the geometrical acceptance of the detector are processed by the vertex reconstruction algorithm. The fast simulation is validated by comparison with the full simulation. The detection efficiencies predicted by the full and the fast simulation differ by less than 5% for all the signal models. The distributions for mass, momentum and transverse momentum of the reconstructed LLP, and for the reconstructed vertex position coincide.

Events with direct production of charm, bottom and top quarks are considered as sources of background. Samples of such events were produced and fully simulated. In particular, $$17 \times 10^6$$ inclusive $${b} {\overline{{b}}} $$ events ($$9 \times 10^6$$ inclusive $${c} {\overline{{c}}} $$ events) were produced with at least two $$b $$ hadrons ($$c $$ hadrons) in $$1.5<\eta <5.0$$, and half a million $${t} {\overline{{t}}} $$ events with at least one muon in the acceptance.

## Event selection and signal determination

This analysis searches for events with pairs of displaced high-multiplicity vertices. The main background is due to secondary interactions of particles with the detector material. These events are discarded by a material veto, which rejects vertices in regions occupied by detector material [23]. The remaining candidates are found to be compatible with ${b} {\overline{{b}}}$ events.

**Fig. 1:**
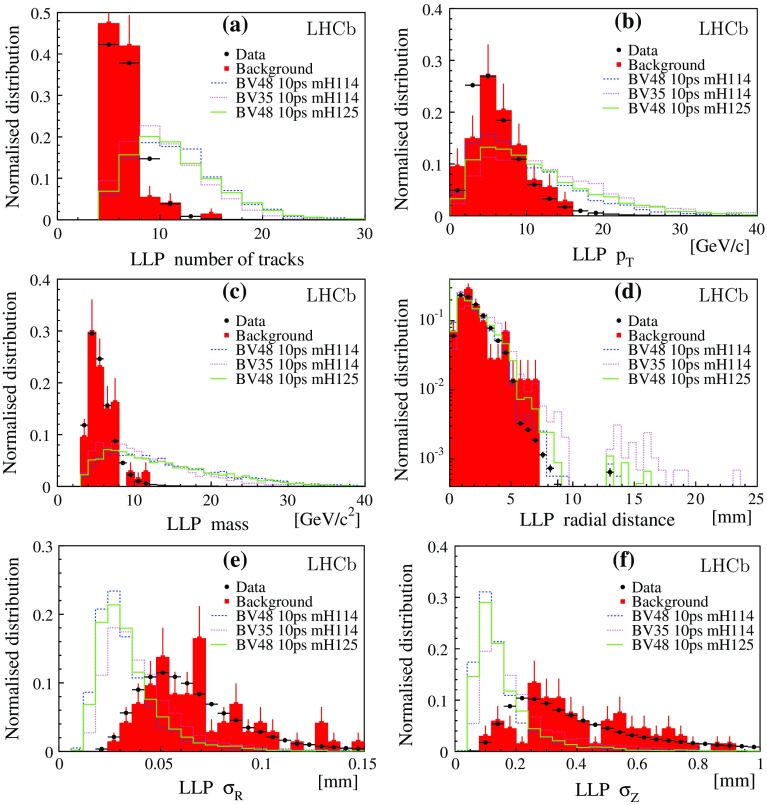
Data (*black dots*) and simulated distributions after preselection normalised to unit integral. There are two LLP candidates per event. The simulated ${b} {\overline{{b}}}$ background is shown by the filled *red histograms* with *error bars*. The *dashed* (*blue*), *dotted* (*purple*) and *solid* (*green*) *lines* are distributions for fully simulated signal models. The subplots show **a** number of tracks used to reconstruct the LLP candidates, **b** LLP transverse momentum, **c** LLP invariant mass, **d** radial distance, $R_\mathrm{xy}$, **e** uncertainty of the radial position, $\sigma _\mathrm{R}$, and **f** uncertainty of the longitudinal position, $\sigma _\mathrm{Z}$, of the LLP vertex

**Fig. 2:**
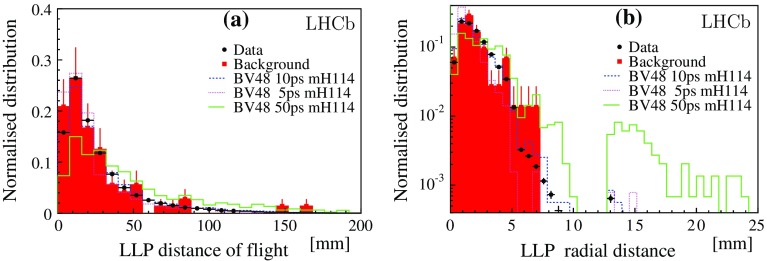
Distributions for **a** the LLP distance of flight from the PV, and, **b** the radial distance of the LLP vertex, $R_\mathrm{xy}$. The fully simulated signal models are chosen with LLP lifetimes of 5, 10, and 50 $\mathrm{ps}$. Symbols are defined as in Fig. 1

From simulation, LLP candidates within the detector acceptance are selected by the L0 and HLT1 triggers with an efficiency of more than 85%. The simulation indicates that the trigger activity is dominated by the hadronic component of the signal expected from high multiplicity events. In HLT2, primary vertices and displaced vertices are reconstructed from charged tracks [24]. Genuine PVs are identified by a small radial distance from the beam axis, $R_\mathrm{xy} <0.3$ mm, and must have at least 10 tracks, including at least one forward track (i.e. in the direction of the spectrometer) and one backward track. Once the set of PVs is identified, all other reconstructed vertices are candidates for the decay position of LLPs. The preselection requires at least one PV in the event and two LLP candidates. The LLP candidates must have at least four forward tracks, no backward tracks, and a minimum invariant mass reconstructed from charged tracks larger than 3.5 ${\mathrm {Ge V\!/}c^2}$ for one candidate, and larger than 4.5 ${\mathrm {Ge V\!/}c^2}$ for the other. In addition, the two secondary vertices must have $R_\mathrm{xy} > 0.4\mathrm \,mm$ and pass the material veto.

The preselection criteria drastically suppress the hadronic background. Only 37 events (74 LLP candidates) survive from the simulated set of $$17.1 \times 10^6$$ $${b} {\overline{{b}}} $$ events generated in the LHCb acceptance, corresponding to an integrated luminosity of 0.3$$ \text{ pb }^{-1}$$. Three simulated $${c} {\overline{{c}}} $$ events pass the selection. They contain $$b $$ hadrons and hence belong to the category of inclusive $${b} {\overline{{b}}} $$, which is also the case of the two surviving $${t} {\overline{{t}}} $$ events. From the 0.62$$ \text{ fb }^{-1}$$ data sample, $$42.9 \times 10^3$$ events are selected. The $${b} {\overline{{b}}} $$ cross-section value measured by LHCb, $$ 288 \pm 4 \pm 48$$ $$\upmu $$b [25, 26], predicts $$(76 \pm 22)\times 10^3$$ events, $$1.8\pm 0.5$$ times the yield observed in data. The estimate uses the next-to-leading-order POWHEG calculation [27] to correct Pythia, and the detection efficiency obtained from the simulated events. The measured yield has also been compared to the rate observed in LHCb by a dedicated inclusive $${b} {\overline{{b}}} $$ analysis, based on a topological trigger [28]. The consistency with the $${b} {\overline{{b}}} $$ background is verified within a statistical precision of 10%.

**Fig. 3:**
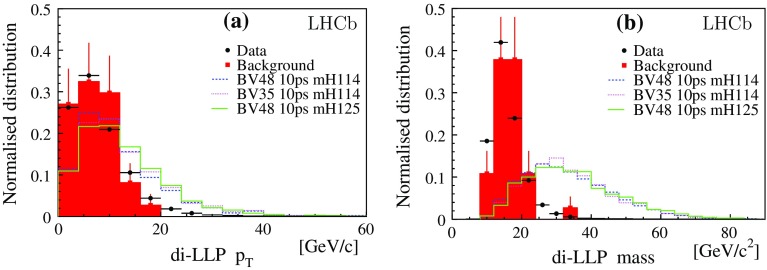
Distributions for **a** the $p_\mathrm{T}$of the Higgs-like candidate, and **b** its invariant mass. Symbols are defined as in Fig. 1

The shapes of the distributions of the relevant observables are compatible with the ${b} {\overline{{b}}}$ background. Figure 1 compares the distributions for the LLP candidates taken from data and from simulated ${b} {\overline{{b}}}$ events. The distributions for three fully simulated signal models are also shown. The mass and the $p_\mathrm{T}$values are calculated assuming the pion mass for each charged track. Figure 1d presents the radial distribution of the displaced vertices; the drop in the number of candidates with a vertex above $R_\mathrm{xy} \sim 5\mathrm \,mm$ is due to the material veto. The variables $\sigma _\mathrm{R}$ and $\sigma _\mathrm{Z}$ shown in Fig. 1e, f are the position uncertainties provided by the vertex fit in the transverse distance $R_\mathrm{xy}$ and along the *z* axis, parallel to the beam. The values of $\sigma _\mathrm{R}$ and $\sigma _\mathrm{Z}$ are larger for the candidates from ${b} {\overline{{b}}}$ background than for the signal because light boosted particles produce close parallel tracks, with the consequence that the vertex fit has larger uncertainties than for the decay of heavier particles producing more diverging tracks. Figure 2 presents the LLP distance of flight and $R_\mathrm{xy}$ distributions compared to three fully simulated signal models, corresponding to $\tau _\mathrm{{LLP}}$ values of 5, 10, and 50 $\mathrm{ps}$.

The reconstructed four-vectors of the two LLPs in the event are added to form the Higgs-like candidate (di-LLP), the corresponding invariant mass and $p_\mathrm{T}$distributions are given in Fig. 3.

Further cuts are applied to the preselected data, to increase the statistical sensitivity. The figure of merit used is given by $\epsilon / \sqrt{N_d + 1}$, where $\epsilon$ is the signal efficiency from simulation for a given selection, and $N_d$ the corresponding number of candidates found in the data. The baseline selection ($\mathrm Sel_1$) is defined by a minimum number of charged tracks on each vertex $N^\mathrm{track}_\mathrm{min} = 6$, a minimum reconstructed mass $m^\mathrm{LLP}_\mathrm{min} =6\,{\mathrm {Ge V\!/}c^2}$, and maximum uncertainties from the vertex fit $\sigma ^\mathrm{R}_\mathrm{max} =0.05 \mathrm \,mm$, and $\sigma ^\mathrm{Z}_\mathrm{max} =0.25\mathrm \,mm$. All the selections used in this analysis are described in Table 1, with the indication of the number of data events selected for a di-LLP reconstructed mass above 19 ${\mathrm {Ge V\!/}c^2}$. Selection $\mathrm Bkg_1$ is used to model the background in the fit procedure described in Sect. 5, selections $\mathrm Sel_2$ and $\mathrm Bkg_2$ are used to study systematic effects.

**Table 1:** Definition of the criteria used for the signal determination. Selections $\mathrm Sel_1$ and $\mathrm B\,kg_1$ are the baseline selections used in the fit, $\mathrm Sel_2$ and $\mathrm B\,kg_2$ are used for the determination of systematic effects. The material veto and the requirement $R_\mathrm{xy} >0.4$ $\mathrm \,mm$ are applied to both LLP candidates. The last column gives the number of data events selected, for a di-LLP reconstructed mass above 19 ${\mathrm {Ge V\!/}c^2}$

Selection	$N^\mathrm{track}_\mathrm{min}$	$m^\mathrm{LLP}_\mathrm{min}$ (${\mathrm {Ge V\!/}c^2}$)	$\sigma ^\mathrm{R}_\mathrm{max}$ ($\mathrm \,mm$)	$\sigma ^\mathrm{Z}_\mathrm{max}$	$N_\mathrm{d}$ ($\mathrm \,mm$)
$\mathrm Sel_1$	6	6	0.05	0.25	157
$\mathrm Sel_2$	5	5	0.05	0.25	387
$\mathrm B\,kg_1$	4	4	–	–	23.2k
$\mathrm B\,kg_2$	5	5	–	–	10.1k

## Determination of the di-LLP signal

The signal yield is determined by a fit of the di-LLP invariant mass, assuming that the two LLPs are the decay products of a narrow resonance. This technique is hampered by the difficulty in producing a reliable background model from simulation, despite the fact that it is reasonable to believe that only ${b} {\overline{{b}}}$ events are the surviving SM component. Therefore, in this analysis the alternative is chosen to infer the background model from data by relaxing the selection requirements, as given by lines $\mathrm Bkg_1$ and $\mathrm Bkg_2$ of Table 1. The comparison of the results obtained with the different signal and background selections is subsequently used to estimate the systematic effects.

**Fig. 4:**
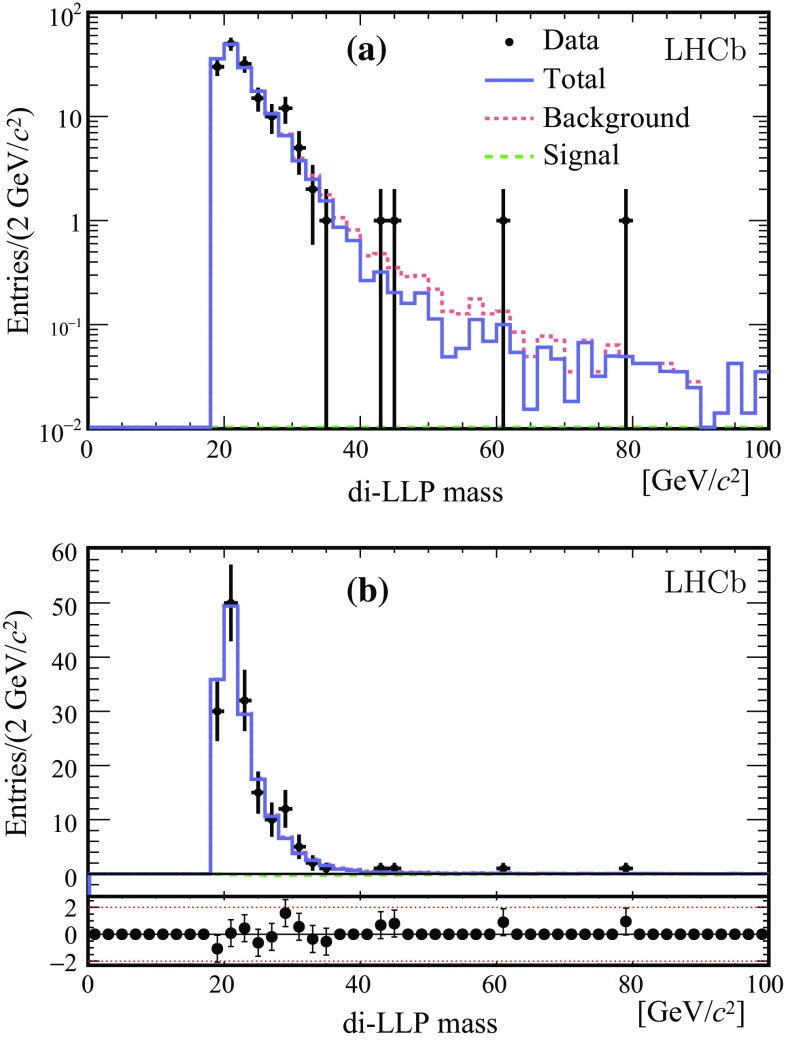
Results of the fit based on the model BV48 10 ps mH114. In **a** log distribution and **b** linear scale with pull distribution. *Dots* with *error bars* are the data, the *dotted* (*red*) and the *dashed* (*green*) *histograms* show the fitted background and signal contributions, respectively. The *purple histogram* is the total fitted distribution

**Fig. 5:**
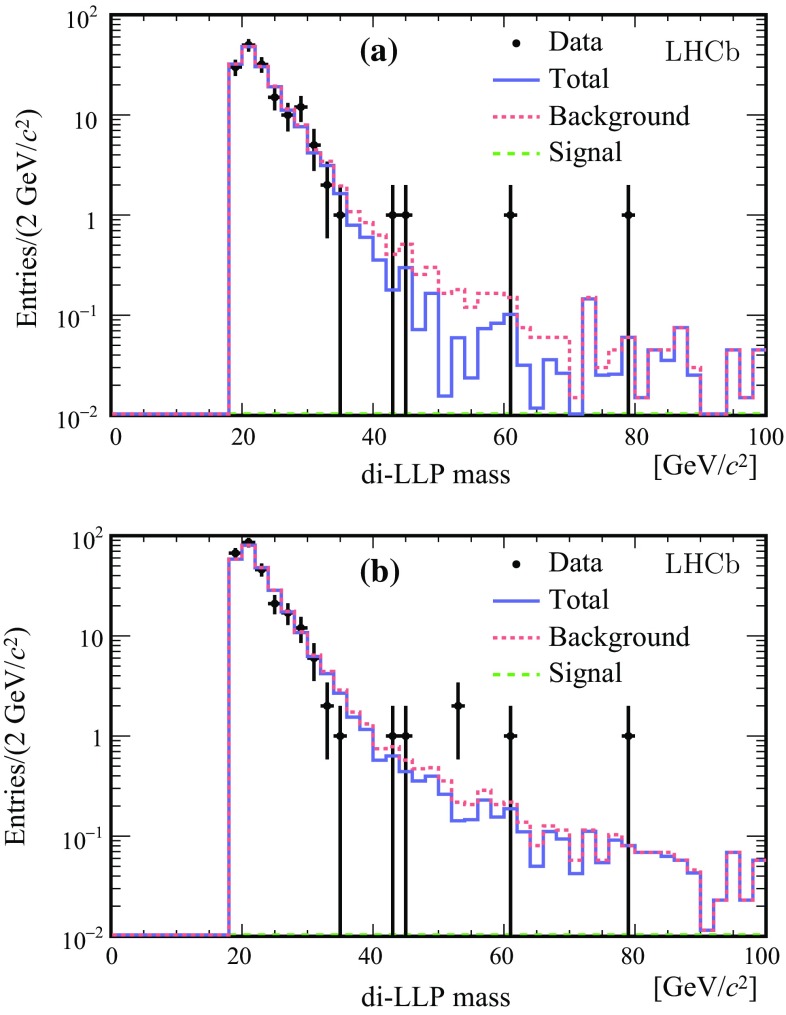
Results of the fit based on the model BV48 10ps mH114, for different combinations of signal and background selections, **a** signal from $\mathrm Sel_1$ and background from $\mathrm B\,kg_2$, **b** signal from $\mathrm Sel_2$ and background from $\mathrm B\,kg_1$. *Dots* with *error bars* are data, the *dashed* (*green*) *line* is the fitted signal and the *dotted* (*red*) *line* the background. In both cases the fitted signal is negative. The *histogram* (*blue*) is the total fitted function

The signal template is the histogram built from BV simulated events selected under the same conditions as data, i.e. $\mathrm Sel_1$. The background template is the histogram obtained from data events selected by the $\mathrm Bkg_1$ conditions. The number of signal (background) candidates $N_s$ ($N_b$) is determined by an extended maximum likelihood fit. The results are given in Fig. 4 for the BV48 10 ps mH114 signal. The fit $\chi ^2/\mathrm {ndf}$ is 0.6. Note that only the portion of the di-LLP mass spectrum above 19 ${\mathrm {Ge V\!/}c^2}$ is used, in order to be sufficiently above the mass threshold set by the selections. Alternatively, $\mathrm Sel_2$ and $\mathrm Bkg_2$ are used to assess systematic effects. The fit results for the selections ($\mathrm Sel_1$,$\mathrm Bkg_2$), ($\mathrm Sel_2$,$\mathrm Bkg_1$) are shown in Fig. 5. The corresponding fit $\chi ^2/\mathrm {ndf}$ values are 0.6 and 1.0. The results are given in Table 2 for all fully simulated signal models. All fits give a negative number of signal candidates, compatible with zero. These results are correlated because the data sample is in common and the di-LLP mass shapes are almost identical for the different fully simulated models as depicted in Fig. 3. A check is performed on 142 di-LLP candidates selected from simulated ${b} {\overline{{b}}}$ background without the requirement on $R_\mathrm{xy}$ and with $m^\mathrm{LLP}_\mathrm{min} =4\,{\mathrm {Ge V\!/}c^2}$ for both LLPs. The fitted number of signal events is $-0.8\pm 3.5$.

**Fig. 6:**
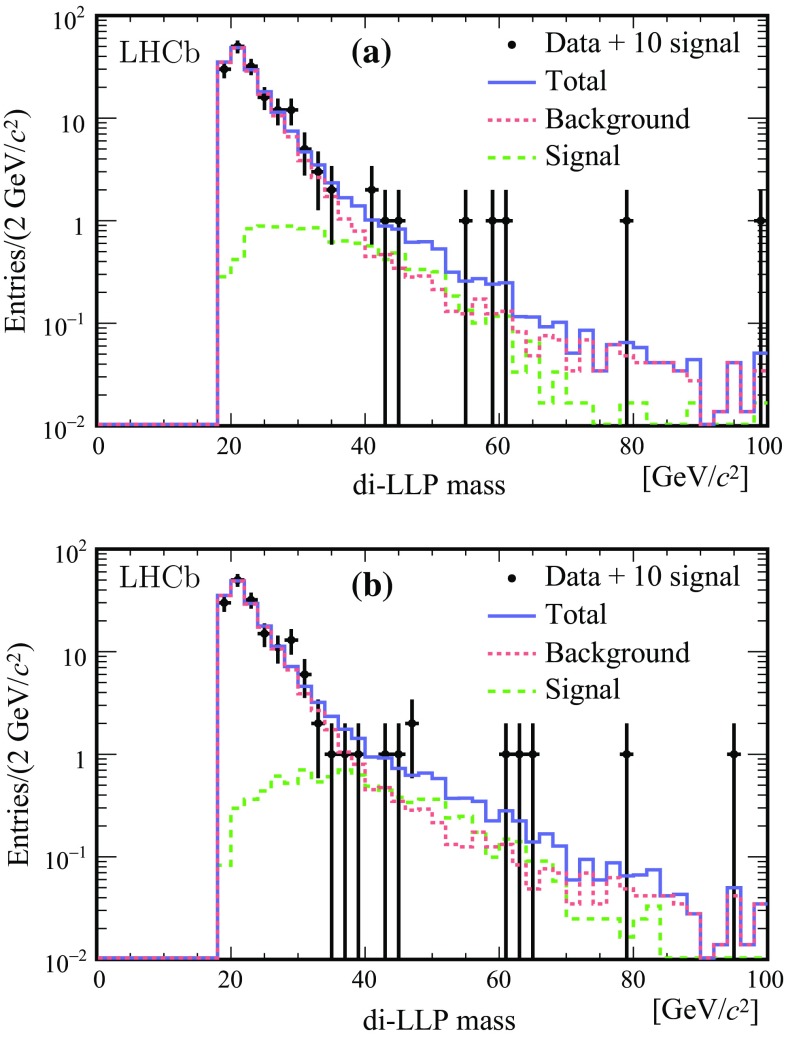
Results of the fit to the data to which 10 signal events have been added randomly chosen following the signal model. For the theoretical model BV48 10 ps mH100, in **a**, the fitted signal is $11.1\pm 7.0$ events; for BV48 10 ps mH125, in **b**, the result is $9.3\pm 5.6$ events

The behaviour and sensitivity of the procedure is further studied by adding a small number of signal events to the data according to a given signal model. Figure 6 shows the results for two models with 10 signal events added to the data. The fitted $N_s$ corresponds well to the number of injected signal events.

**Table 2:** Values of the fitted signal and background events for the different fully simulated signal models. The signal/background combinations are defined in the first row

Model	($\mathrm Sel_1$, $\mathrm B\,kg_1$)		($\mathrm Sel_1$, $\mathrm B\,kg_2$)	($\mathrm Sel_2$, $\mathrm B\,kg_1$)
	$N_s$	$N_b$	$N_s$	$N_s$
BV48 5ps mH114	$-2.6 \pm 4.4$	163.6 $\pm \, 13.6$	$-4.8 \pm 3.9$	$-1.7\pm 3.9$
BV48 10ps mH114	$-3.3 \pm 3.5$	164.3 $\pm \, 13.4$	$-4.6 \pm 3.1$	$-3.1\pm 3.6$
BV48 15ps mH114	$-3.5 \pm 3.6$	164.5 $\pm \, 13.5$	$-4.4 \pm 3.1$	$-2.0\pm 3.6$
BV48 50ps mH114	$-1.4 \pm 3.6$	162.4 $\pm \, 13.3$	$-2.7 \pm 3.4$	$-2.1\pm 4.2$
BV48 100ps mH114	$-0.7 \pm 4.1$	161.7 $\pm \, 13.4$	$-3.5 \pm 3.9$	$-3.2\pm 4.2$
BV35 10ps mH114	$-4.3 \pm 3.3$	165.3 $\pm \, 13.4$	$-5.9 \pm 3.1$	$-4.6\pm 3.5$
BV20 10ps mH114	$-1.9 \pm 1.6$	162.8 $\pm \, 12.9$	$-2.7 \pm 1.7$	$-2.0\pm 2.4$
BV48 10ps mH100	$-1.7 \pm 4.7$	162.7 $\pm \, 13.7$	$-4.4 \pm 4.4$	$-5.2\pm 4.7$
BV48 10ps mH125	$-2.8 \pm 3.5$	163.8 $\pm \, 13.4$	$-4.1 \pm 3.2$	$-3.2\pm 3.6$
BV55 10ps mH114	$-3.1 \pm 3.7$	164.1 $\pm \, 13.5$	$-4.6 \pm 3.4$	$-1.1\pm 3.7$
BV55 10ps mH125	$-2.6 \pm 3.5$	163.6 $\pm \, 13.4$	$-4.0 \pm 3.2$	$-3.9\pm 3.8$

An alternative fit procedure has been applied, using parameterised signal and background templates. The sum of two exponential functions is used for the background, and an exponential convolved with a Gaussian function for the signal. The results are consistent with a null signal for all the models.

As a final check a two-dimensional sideband subtraction method (“ABCD method” [29]) has been applied in the reconstructed mass of one LLP and the number of tracks of the other LLP, also giving results consistent with zero signal.

## Detection efficiency and systematic uncertainties

The determination of the detection efficiency is based on simulated events. The geometrical acceptance for the detection of one $\tilde{\chi }^{0}_{1}$ in LHCb is, depending on the model, between 20 and 30%. After selection $\mathrm Sel_1$ the predicted total di-LLP detection efficiency is in the range 0.1–1% for most of the models. Potential discrepancies between simulation and data are considered as sources of systematic uncertainties. Table 3 summarises the contributions of the systematic uncertainties, which are valid for all fully simulated models, dominated by the 15% contribution from the trigger.

**Table 3:** Contributions to the systematic uncertainty for fully simulated models. For the analysis based on the fast simulation the same total systematic uncertainty is adopted augmented by 5% to account for the relative imprecision of the fast and full simulations. The contributions from the signal and the data-driven background models used in the di-LLP mass fit are discussed in the text

Source	%
Trigger	15
Track reconstruction	5
Vertex reconstruction	4
$p_\mathrm{T}$ and mass calibration	6
Material veto	4
PV multiplicity	0.1
Beam line position	0.7
Theoretical model	9.9
Integrated luminosity	$1.7$
Total	$20.5$

The consistency between the trigger efficiency in data and simulation is checked by selecting LLP events with an independent trigger, designed for the detection of ${J /\psi }$ events. Comparing the fraction of the data that also passes the double-LLP selection with the corresponding fraction in simulated inclusive ${J /\psi }$ events, consistent efficiencies are found within a statistical uncertainty of 30%. A more precise result is obtained when requiring only a single LLP candidate [9] and assuming uncorrelated contributions from the two LLPs to determine the efficiency for detecting two LLPs in coincidence. A maximum discrepancy between data and simulation of 15% is inferred, which is the value adopted.

The consistency between the track reconstruction efficiency in data and simulation is studied by a comparison of the number of tracks selected in displaced vertices from ${b} {\overline{{b}}}$ events. The average number of tracks per LLP in data is higher than in simulated events by about 0.07 tracks. Assuming that this small effect is entirely due to a difference in tracking efficiency, the overall di-LLP detection efficiency changes by at most 5%.

**Table 4:** Detection efficiency with total uncertainty, and upper limits at 95% CL on the cross-section times branching ratio for the process $pp \rightarrow \mathrm {h}^0X$, $\mathrm {h}^0\rightarrow \tilde{\chi }^{0}_{1} \tilde{\chi }^{0}_{1} \rightarrow 6q$ for the fully simulated models

Model	Efficiency (%)	Expected upper limit ($\mathrm pb$)	Observed upper limit ($\mathrm pb$)
BV48 5ps mH114	0.528 ± 0.114	3.2$^{+ 2.1}_{- 1.1}$	3.5
BV48 10ps mH114	0.925 ± 0.194	1.8$^{+ 1.2}_{- 0.6}$	1.7
BV48 15ps mH114	0.966 ± 0.208	1.8$^{+ 1.2}_{- 0.6}$	1.6
BV48 50ps mH114	0.419 ± 0.090	4.6$^{+ 2.9}_{- 1.6}$	4.4
BV48 100ps mH114	0.171 ± 0.037	11.9$^{+ 7.1}_{- 3.8}$	12.3
BV35 10ps mH114	0.268 ± 0.058	5.6$^{+ 3.8}_{- 2.0}$	4.9
BV20 10ps mH114	0.016 ± 0.003	52$^{+ 38}_{- 20}$	54
BV48 10ps mH100	0.864 ± 0.186	2.5$^{+ 1.6}_{- 0.8}$	2.6
BV48 10ps mH125	0.771 ± 0.166	2.0$^{+ 1.4}_{- 0.7}$	2.0
BV55 10ps mH114	0.851 ± 0.183	1.9$^{+ 1.3}_{- 0.7}$	1.9
BV55 10ps mH125	0.937 ± 0.201	1.7$^{+ 1.1}_{- 0.6}$	1.7

The vertex reconstruction efficiency is affected by the tracking efficiency and resolution. A study of vertices from $B^0 \rightarrow {{J /\psi }} {{K} ^{*0}}$ with ${{J /\psi }} \!\rightarrow {\mu ^{+}\mu ^{-}}$ and ${{K} ^{*0}} \rightarrow K^{+} \pi ^{-}$ has shown that the data and simulation detection efficiencies for this four-prong process agree within 7.5% [9]. This has been evaluated to correspond at most to a 4% discrepancy between the di-LLP efficiency in data and simulation.

A maximum mismatch of 10% on both the transverse momentum and mass scales is inferred from the comparison of data and simulated ${b} {\overline{{b}}}$ distributions, which propagates to a 6% contribution to the systematic uncertainty.

The effect of the material veto corresponds to a reduction of the geometrical acceptance and depends mainly on the LLP lifetime. An analysis with the requirement of $R_\mathrm{xy} < 4\mathrm \,mm$ allows to infer a maximum systematic uncertainty of 4%.

A small contribution to the systematic uncertainty of 0.1% is determined by reweighting the simulated events to match the PV multiplicity in the data.

The uncertainty on the position of the beam line is less than 20${\,\upmu \mathrm m}$  [30]. It can affect the secondary vertex selection, mainly via the requirement on $R_\mathrm{xy}$. By altering the PV position in simulated signal events, the maximum effect on the di-LLP selection efficiency is 0.7%.

The Higgs-like particle production model is mainly affected by the uncertainty on the parton luminosity. A maximum variation of the detection efficiency of 9.5% is obtained following the prescriptions given in [31]. A second contribution of 3% is obtained by reweighting the Pythia generated events to match a recent calculation of the $p_\mathrm{T}$distributions [32]. The total theoretical uncertainty is 9.9%, obtained by summing in quadrature the mentioned contributions.

In addition to the systematic uncertainty on the detection efficiency, the following contributions have been considered. The uncertainty on the integrated luminosity is 1.7% [33]. As previously stated, the uncertainty on the momentum scale and the invariant mass scale is smaller than 10%. This value is also assumed for the di-LLP mass calibration. To assess the impact on the signal measurement, pseudoexperiments are produced with 10 events of simulated signal added to the background following the nominal signal distribution but with the di-LLP mass value scaled by $\pm 10\%$. The subsequent maximum variation of the fitted number of events is $\pm 1.6$, for all the signal hypotheses. The uncertainty due to the shape of the background template is obtained by comparing the number of fitted events obtained with the $\mathrm B\,kg_1$ and $\mathrm B\,kg_2$ selections. The change is less than one event, for all the signal models. The difference in data and simulation in the di-LLP mass resolution and the statistical precision of the signal templates used in the fit have a negligible effect. Hence, a fit uncertainty of ±2 events is considered in the calculation of the cross-section upper limits.

For the analysis based on the fast simulation, a 5% uncertainty is added to account for the relative imprecision of the fast simulation with respect to the full simulation, as explained in Sect. 3.

## Results

**Fig. 7:**
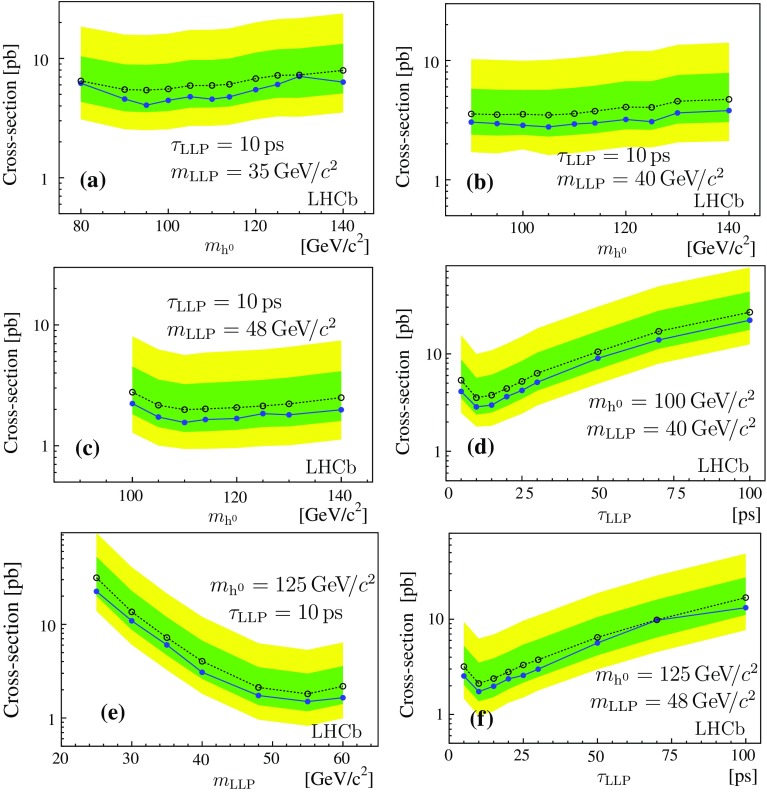
Expected (*open dots* with 1$\sigma$ and 2$\sigma$ bands) and observed (*full dots*) upper limits at 95% confidence level, **a**–**c** shown for different masses of the Higgs-like particle, **d**, **f** for different LLP lifetimes, and **e** as a function of the LLP mass. The values of the other parameters are indicated on the plots. Results inferred from the fast simulation

The 95% confidence level (CL) upper limits on the production cross-section times branching ratio are presented in Table 4, for the fully simulated models, based on the CLs approach [34]. The fast simulation allows the exploration of a larger region of parameter space. The cross-section times branching fraction upper limits at 95% CL for benchmark theoretical models are shown in Fig. 7 (the corresponding tables are given in Appendix C).

The estimated detection efficiencies can be found in Appendix B, Tables 6 and 7. The efficiency increases with $$m_\mathrm{{LLP}}$$ because more particles are produced in the decay of heavier LLPs. This effect is only partially counteracted by the loss of particles outside of the spectrometer acceptance, which is especially the case with heavier Higgs-like particles. Another competing phenomenon is that the lower boost of heavier LLPs results in a shorter average flight length, i.e. the requirement of a minimum $$R_\mathrm{xy}$$ disfavours heavy LLPs. The cut on $$R_\mathrm{xy}$$ is more efficient at selecting LLPs with large lifetimes, but for lifetimes larger than $$\sim $$ $$50\,\mathrm{ps} $$ a portion of the decays falls into the material region and is discarded. Finally, a drop of sensitivity is expected for LLPs with a lifetime close to the $$b $$ hadron lifetimes, where the contamination from $${b} {\overline{{b}}} $$ events becomes important, especially for low mass LLPs.

## Conclusion

A search for Higgs-like bosons decaying into two long-lived particles decaying hadronically has been carried out using data from *pp* collisions at 7$\mathrm {\,Te V}$ collected with the LHCb detector, corresponding to a total integrated luminosity of $0.62$ $\text{ fb }^{-1}$.

The model used to describe the LLP decay is an mSUGRA process in which the lightest neutralino $\tilde{\chi }^{0}_{1}$ decays through a baryon number violating coupling to three quarks. Upper limits have been placed on the production cross-section for Higgs-like boson masses from 80 to 140 ${\mathrm {Ge V\!/}c^2}$, LLP masses in the range 20–60 ${\mathrm {Ge V\!/}c^2}$, and LLP lifetimes in the range of 5–100 $\mathrm{ps}$. The number of candidates is determined by the di-LLP invariant mass fit with signal templates inferred from simulation, and background estimates from data. For the explored parameter space of the theory all results, which are correlated, are consistent with zero. Upper limits at 95% CL for cross-section times branching ratio of 1 to 5$\mathrm pb$ are inferred for most of the considered parameter range. They are below 2$\mathrm pb$ for the decay of a 125 ${\mathrm {Ge V\!/}c^2}$ Higgs-like particle in two LLPs with mass in the 48–60 ${\mathrm {Ge V\!/}c^2}$ range and 10 $\mathrm{ps}$ lifetime.

## Fully simulated signal datasets

Table 5 shows the parameters used to generate the 11 fully simulated signal models with Pythia  6. The Higgs-like boson is produced by gluon-gluon fusion. In the table M$_1$ corresponds to the Pythia parameter RMSS(1), and $\tan {\beta }$  to RMSS(5). In addition, M$_2$ (RMSS(2)) is set at 250 ${\mathrm {Ge V\!/}c^2}$ and $\mu$ (RMSS(4)) has the value 140. A $m_\mathrm{h^0}$ value of 125 ${\mathrm {Ge V\!/}c^2}$ requires RMSS(16) = 2300.

**Table 5:** Parameters of the signal models generated by Pythia and fully simulated

Model	M$_1$ (${\mathrm {Ge V\!/}c^2}$)	$\tan {\beta }$	$m_\mathrm{h^0}$ (${\mathrm {Ge V\!/}c^2}$)	$m_\mathrm{{LLP}}$ (${\mathrm {Ge V\!/}c^2}$)	$\tau _\mathrm{{LLP}}$ ($\mathrm{ps}$)
BV48 5ps mH114	62	5	114	48	5
BV48 10ps mH114	62	5	114	48	10
BV48 15ps mH114	62	5	114	48	15
BV48 50ps mH114	62	5	114	48	50
BV48 100ps mH114	62	5	114	48	100
BV35 10ps mH114	46	5	114	35	10
BV20 10ps mH114	28	5	114	20	10
BV48 10ps mH100	71	2.4	100	48	10
BV48 10ps mH125	60	8	125	48	10
BV55 10ps mH114	71	5.1	114	55	10
BV55 10ps mH125	69	6.2	125	55	10

## Detection efficiencies

Table 6 gives the detection efficiency as a function of $m_\mathrm{h^0}$ and $m_\mathrm{{LLP}}$, the LLP lifetime is 10 $\mathrm{ps}$. Table 7 gives the efficiency as a function of $m_\mathrm{{LLP}}$ and $\tau _\mathrm{{LLP}}$, assuming $m_\mathrm{h^0} =114\,{\mathrm {Ge V\!/}c^2}$.

**Table 6:** Detection efficiency values in percent estimated by the fast simulation as a function of $m_\mathrm{h^0}$ and $m_\mathrm{{LLP}}$. The LLP lifetime is 10 ps. The statistical uncertainty is 10% for $\epsilon \sim 0.02\%$, 5 % for $\epsilon \sim 0.1\%$, 3% for $\epsilon \sim 0.5\%$, and 2% for $\epsilon \sim 1\%$

$m_\mathrm{h^0}$ $\mathrm{(GeV/c^2)}$	$m_\mathrm{{LLP}}$ (GeV/c$^2$)							
	20	25	30	35	40	48	55	60
80	0.035	0.126	0.276	0.514	–	–	–	–
90	0.027	0.084	0.213	0.456	0.699	–	–	–
95	0.023	0.077	0.203	0.414	0.689	–	–	–
100	0.025	0.073	0.184	0.368	0.647	0.858	–	–
105	0.018	0.066	0.139	0.324	0.574	1.018	–	–
110	0.017	0.053	0.146	0.291	0.525	1.016	–	–
114	0.014	0.048	0.134	0.259	0.472	0.963	0.817	–
120	0.016	0.047	0.107	0.222	0.402	0.836	1.013	–
125	0.009	0.042	0.097	0.225	0.377	0.765	0.997	–
130	0.014	0.037	0.085	0.191	0.325	0.708	0.914	0.991
140	0.002	0.031	0.075	0.163	0.277	0.566	0.782	0.881

**Table 7:** Detection efficiency in percent estimated by the fast simulation as a function of the $m_\mathrm{{LLP}}$ and $\tau _\mathrm{{LLP}}$, for $m_\mathrm{h^0}$ = 114 GeV/c$^2$. The statistical uncertainty is 10% for $\epsilon \sim 0.02\%$, 5 % for $\epsilon \sim 0.1\%$, 3% for $\epsilon \sim 0.5\%$, and 2% for $\epsilon \sim 1\%$

$\tau _\mathrm{{LLP}}$ $\mathrm{(ps)}$	$m_\mathrm{{LLP}}$ (GeV/c$^2$)						
	20	25	30	35	40	48	55
5	0.021	0.053	0.129	0.234	0.366	0.545	0.289
10	0.014	0.048	0.134	0.259	0.472	0.963	0.817
15	0.013	0.042	0.113	0.198	0.389	0.932	1.052
20	0.007	0.035	0.083	0.174	0.338	0.834	1.150
25	0.006	0.034	0.073	0.148	0.289	0.731	1.126
30	0.005	0.026	0.066	0.128	0.241	0.643	1.091
40	0.003	0.017	0.044	0.114	0.193	0.490	0.960
50	0.004	0.015	0.035	0.082	0.157	0.397	0.806
70	0.002	0.009	0.021	0.062	0.104	0.280	0.596
100	0.001	0.005	0.015	0.033	0.071	0.178	0.383

## Cross-section upper limits tables

Expected and observed 95% CL cross-section times branching ratio upper limits for benchmark models, from the fast simulation. Tables 8 and 9 give the limits as a function of $m_\mathrm{h^0}$, covering LLP masses from 35 to 60 ${\mathrm {Ge V\!/}c^2}$, $\tau _\mathrm{{LLP}} =10\,\mathrm{ps}$. Table 10: limits as a function of the LLP lifetime for $m_\mathrm{h^0} =100\,{\mathrm {Ge V\!/}c^2}$ and $m_\mathrm{{LLP}} =40\,{\mathrm {Ge V\!/}c^2}$, and for $m_\mathrm{h^0} =125\,{\mathrm {Ge V\!/}c^2}$ and $m_\mathrm{{LLP}} =48\,{\mathrm {Ge V\!/}c^2}$. Table 11: limits as a function of the LLP mass, for $m_\mathrm{h^0} =125\,{\mathrm {Ge V\!/}c^2}$, $\tau _\mathrm{{LLP}} =10\,\mathrm{ps}$.

**Table 8:** Expected and observed 95% CL cross-section times branching ratio upper limits as a function of $m_\mathrm{h^0}$, with $m_\mathrm{{LLP}} =35\,{\mathrm {Ge V\!/}c^2}$, and $\tau _\mathrm{{LLP}} =10\,\mathrm{ps}$, estimated by the fast simulation

Model	Expected upper limit (pb)	Observed upper limit (pb)
BV35 10ps mH80	6.49$^{+ 3.94}_{- 2.16}$	6.20
BV35 10ps mH90	5.50$^{+ 3.42}_{- 1.89}$	4.56
BV35 10ps mH95	5.42$^{+ 3.41}_{- 1.88}$	4.06
BV35 10ps mH100	5.55$^{+ 3.52}_{- 1.92}$	4.45
BV35 10ps mH105	5.92$^{+ 3.79}_{- 2.06}$	4.78
BV35 10ps mH110	5.94$^{+ 3.79}_{- 2.06}$	4.56
BV35 10ps mH114	6.07$^{+ 3.92}_{- 2.11}$	4.77
BV35 10ps mH120	6.79$^{+ 4.42}_{- 2.39}$	5.47
BV35 10ps mH125	7.21$^{+ 4.70}_{- 2.54}$	6.03
BV35 10ps mH130	7.28$^{+ 4.83}_{- 2.59}$	7.08
BV35 10ps mH140	7.95$^{+ 5.32}_{- 2.85}$	6.35

**Table 9:** Expected and observed 95% CL cross-section times branching ratio upper limits as a function of $m_\mathrm{h^0}$, for LLP masses of 40, 48, 55, and 60 ${\mathrm {Ge V\!/}c^2}$, $\tau _\mathrm{{LLP}} =10\,\mathrm{ps}$, estimated by the fast simulation

Model	Expected upper limit (pb)	Observed upper limit (pb)
BV40 10ps mH90	3.57$^{+ 2.23}_{- 1.18}$	3.04
BV40 10ps mH95	3.52$^{+ 2.18}_{- 1.17}$	2.96
BV40 10ps mH100	3.55$^{+ 2.12}_{- 1.16}$	2.86
BV40 10ps mH105	3.49$^{+ 2.19}_{- 1.18}$	2.77
BV40 10ps mH110	3.59$^{+ 2.32}_{- 1.21}$	2.93
BV40 10ps mH114	3.76$^{+ 2.38}_{- 1.30}$	2.99
BV40 10ps mH120	4.07$^{+ 2.63}_{- 1.42}$	3.20
BV40 10ps mH125	4.04$^{+ 2.66}_{- 1.43}$	3.07
BV40 10ps mH130	4.55$^{+ 2.98}_{- 1.61}$	3.63
BV40 10ps mH140	4.71$^{+ 3.14}_{- 1.69}$	3.79
BV48 10ps mH100	2.78$^{+ 1.75}_{- 0.95}$	2.23
BV48 10ps mH105	2.17$^{+ 1.36}_{- 0.74}$	1.73
BV48 10ps mH110	1.99$^{+ 1.24}_{- 0.69}$	1.56
BV48 10ps mH114	2.02$^{+ 1.29}_{- 0.70}$	1.65
BV48 10ps mH120	2.07$^{+ 1.34}_{- 0.71}$	1.68
BV48 10ps mH125	2.12$^{+ 1.38}_{- 0.74}$	1.74
BV48 10ps mH130	2.22$^{+ 1.45}_{- 0.78}$	1.80
BV48 10ps mH140	2.49$^{+ 1.65}_{- 0.89}$	1.98
BV55 10ps mH130	1.94$^{+ 1.27}_{- 0.69}$	1.76
BV55 10ps mH140	1.93$^{+ 1.26}_{- 0.69}$	1.75
BV60 10ps mH130	1.79$^{+ 1.16}_{- 0.63}$	1.52
BV60 10ps mH140	1.86$^{+ 1.21}_{- 0.66}$	1.48

**Table 10:** Expected and observed 95% CL cross-section times branching ratio upper limits as a function of the LLP lifetime, for $m_\mathrm{h^0} = 100\,{\mathrm {Ge V\!/}c^2}$ and $m_\mathrm{{LLP}} =40\,{\mathrm {Ge V\!/}c^2}$, and for $m_\mathrm{h^0} = 125\,{\mathrm {Ge V\!/}c^2}$ and $m_\mathrm{{LLP}} =48\,{\mathrm {Ge V\!/}c^2}$, estimated by the fast simulation

Model	Expected upper limit (pb)	Observed upper limit (pb)
BV40 5ps mH100	5.36$^{+ 3.36}_{- 1.85}$	4.11
BV40 10ps mH100	3.55$^{+ 2.12}_{- 1.16}$	2.86
BV40 15ps mH100	3.76$^{+ 2.34}_{- 1.26}$	2.98
BV40 20ps mH100	4.41$^{+ 2.73}_{- 1.49}$	3.63
BV40 25ps mH100	5.21$^{+ 3.23}_{- 1.75}$	4.20
BV40 30ps mH100	6.32$^{+ 3.95}_{- 2.13}$	5.10
BV40 50ps mH100	10.5$^{+ 6.5}_{- 3.6}$	9.0
BV40 70ps mH100	17.0$^{+ 10.6}_{- 5.8}$	13.8
BV40 100ps mH100	26.7$^{+ 16.5}_{- 9.1}$	22.1
BV48 5ps mH125	3.19$^{+ 2.06}_{- 1.14}$	2.54
BV48 10ps mH125	2.12$^{+ 1.38}_{- 0.74}$	1.74
BV48 15ps mH125	2.38$^{+ 1.50}_{- 0.86}$	1.98
BV48 20ps mH125	2.80$^{+ 1.76}_{- 0.95}$	2.37
BV48 25ps mH125	3.31$^{+ 2.11}_{- 1.15}$	2.57
BV48 30ps mH125	3.76$^{+ 2.38}_{- 1.28}$	2.99
BV48 50ps mH125	6.45$^{+ 4.09}_{- 2.26}$	5.63
BV48 70ps mH125	9.86$^{+ 6.23}_{- 3.42}$	9.74
BV48 100ps mH125	16.9$^{+ 10.6}_{- 5.8}$	13.2

**Table 11:** Expected and observed 95% CL cross-section times branching ratio upper limits as a function of the LLP mass, with $m_\mathrm{h^0} =125\,{\mathrm {Ge V\!/}c^2}$ and $\tau _\mathrm{{LLP}} =10\,\mathrm{ps}$, estimated by the fast simulation

Model	Expected upper limit (pb)	Observed upper limit (pb)
BV20 10ps mH125	95.3$^{+ 64.9}_{- 34.7}$	112.6
BV25 10ps mH125	31.4$^{+ 21.0}_{- 11.3}$	22.5
BV30 10ps mH125	13.6$^{+ 9.1}_{- 4.9}$	10.9
BV35 10ps mH125	7.21$^{+ 4.70}_{- 2.54}$	6.03
BV40 10ps mH125	4.04$^{+ 2.66}_{- 1.43}$	3.07
BV48 10ps mH125	2.12$^{+ 1.38}_{- 0.74}$	1.74
BV55 10ps mH125	1.81$^{+ 1.17}_{- 0.63}$	1.50
BV60 10ps mH125	2.18$^{+ 1.40}_{- 0.76}$	1.64
